# Plant Diseases and Sustainable Agriculture

**DOI:** 10.3390/plants14081175

**Published:** 2025-04-10

**Authors:** Patrick Materatski, Cláudia S. L. Vicente, Carla Varanda, Margarida Espada

**Affiliations:** 1 MED—Mediterranean Institute for Agriculture, Environment and Development & CHANGE—Global Change and Sustainability Institute, Institute for Advanced Studies and Research, University of Évora, Pólo da Mitra, 7006-554 Évora, Portugal; pmateratski@uevora.pt (P.M.); cvicente@uevora.pt (C.S.L.V.); carla.varanda@esa.ipsantarem.pt (C.V.); 2 Research Centre for Natural Resources, Environment and Society (CERNAS), Santarém Polytechnic University, School of Agriculture, Quinta do Galinheiro—S. Pedro, 2001-904 Santarém, Portugal

## 1. Introduction

Plant pathogens including viruses, bacteria, fungi, nematodes, and insects, can cause huge economic losses on a wide range of crops and forest species worldwide. At present, we face several challenges such as climate change, population growth, and limited resources [1]. Sustainability strategies worldwide (e.g., the European Green Deal under the Farm to Fork and Biodiversity Strategies and ONU Sustainable Development Goals) emphasize the importance of a resilient agri-food sector and an efficient use of natural resources. To meet these challenges, plant protection represents a key factor in developing efficient disease management practices. Plant pathogens employ different pathogenic strategies to overcome plant defenses. For the success of plant protection, it is essential to be one step ahead of these pathogens. This is possible by understanding how these agents interact with their hosts, vectors, other pathogens, and the environment. We must also predict how all these drivers will respond to climate change, which can increase disease severity and lead to pathogens spreading into new areas.

In this Special Issue ‘Plant Diseases and Sustainable Agriculture’, we have gathered up-to-date research on the importance of the study of plant pathogens to achieve sustainable agriculture, reinforcing that the more we know them in terms of biology, epidemiology, and genetics, the more we will be prepared to control them in a sustainable manner.

## 2. Special Issue Overview

This Special Issue compiles five articles which show innovative research in the study of different plant pathogens towards sustainable agriculture. These five contributions are listed below.

The use of artificial intelligence in agriculture is now a useful tool in field conditions. Wang et al., 2024 (contribution 1) aimed to address the challenge of disease similarity caused by seven common apple leaf diseases, by proposing an optimized RegNet model to identify the pathogens. The study evaluates several factors like training methods, data expansion, optimizer selection, and image background. The results show that offline data expansion and transfer learning improve classification performance, while complex image backgrounds reduce accuracy. The optimized RegNet model achieved testing accuracies of 93.85% and 99.23% for two datasets, demonstrating its potential for accurate disease identification in apple orchards under challenging field conditions. In the same line of research, Wan et al., 2024 (contribution 2), focused on the identification of pepper diseases, such as blight and anthracnose, which are a major threat to crops, leading to yield and economic losses. The study proposed a new recognition model—TPSAO-AMWNet—to address challenges like edge-blurring and extraction of small features in disease images. The TPSAO-AMWNet model achieved an average accuracy of 93.52% and a F1 score of 93.15%, proving effective in classifying pepper diseases. These results also offer valuable insights into disease detection in other crops. Also in pepper crop, Mensah et al., 2024 (contribution 3), addressed the challenge of detecting *Tomato spotted wilt virus* (TSWV), without using traditional methods such as symptom observation or molecular techniques, such as reverse transcription-PCR, which are time-consuming and sometimes inconclusive. This study compared two imaging techniques—RGB (Red–Green–Blue) and hyperspectral imaging with a bioassay method to detect TSWV incidence and severity in pepper with different levels of susceptibility to TSWV. With this study, authors were able to show that high-resolution RGB images can be used to identify symptoms and that the same regions can be analyzed through hyperspectral methods, providing a reliable method for detecting disease spots with higher accuracy, offering a faster and more precise alternative method to the traditional ones.

Understanding and mitigating the effects of plant pathogens is a critical research area within plant protection, which encompasses all strategies aiming to develop solutions to protect plants from pests, diseases, and environmental/abiotic factors. Plant-parasitic nematodes (PPNs) can significantly reduce crop yields, affecting economically important agricultural and forestry species worldwide, representing over 100 billion dollars globally [2]. Within the top 10 most damaging PPNs [3], the migratory endoparasitic pinewood nematode *Bursaphelenchus xylophilus* is the only PPNs responsible for affecting forests ecosystems, causing the pine wilt disease. Efforts have been made to study their biology and the molecular mechanisms of the nematode pathogenicity in interaction with the host to develop new sustainable control strategies to promote plant resistance, minimizing the use of pesticides. Mendonça et al. (2024) (contribution 4) studied the role of ShK-domain-like proteins, which may increase our knowledge of how nematodes modulate hosts during infection and develop new target molecules for nematode control potentially applied in plant biotechnology field. ShK domain-containing proteins are 35/37-residue peptide toxin capable of blocking the potassium channels. In the pinewood nematode (*B. xylophilus*), most of the ShK transcripts were highly expressed during infection [4] and upregulated in response to reactive oxygen species (ROS) products (hydrogen peroxide). The study suggested, for the first time, that nematode’s ShK domain proteins may be involved in managing oxidative stress, potentially protecting or modulating ROS activity in the host plants during parasitism.

On a different approach, Faria and Barbosa (2024) (contribution 5) explored the potential of plant essential oils (EOs) to develop sustainable alternatives to traditional pesticides. EOs extracted through simple distillation from plant material are promising due to their strong biological activity and diverse chemical composition. The EOs of *Cymbopogon citratus* revealed significant nematicidal activity against the pinewood nematode with higher activity linked to oxygen-containing compounds like citral and geraniol, compared to monoterpene hydrocarbons like β-myrcene. The oxygen-containing compounds in the EOs, particularly citral and geraniol, were found to be highly effective against pinewood nematode and less toxic to non-target organisms compared to traditional nematicides. Despite their advantages, the use of EOs is still limited due to regulatory challenges, stability issues, and inconsistent EO quality. However, the promising results suggest that plant-based EOs could serve as sustainable and safer alternatives to conventional pesticides.

## 3. Concluding Remarks

The manuscripts featured in this Special Issue encompass studies on a wide range of plant pathogens, offering invaluable knowledge to science. As Guest Editors of this Special Issue “Plant Diseases and Sustainable Agriculture”, we would like to thank all the authors for submitting such interesting contributions. It has been a pleasure to read and learn from their works. We would also like to thank the reviewers for their valuable comments on the manuscripts, as well as the Editorial Office for all their support.

## References

[1] Savary S., Willocquet L., Pethybridge S.J., Esker P., McRoberts N., Nelson A. (2019). The global burden of pathogens and pests on major foodcrops. Nat. Ecol. Evol..

[2] Kantor M., Handoo Z., Kantor C., Carta L. (2022). Top Ten Most Important U.S.-Regulated and Emerging Plant-Parasitic Nematodes. Horticulturae.

[3] Jones J.T., Haegeman A., Danchin E.G., Gaur H.S., Helder J., Jones M.G., Kikuchi T., Manzanilla-López R., Palomares-Rius J.E., Wesemael W.M. (2013). Top 10 plant-parasitic nematodes in molecular plant pathology. Mol. Plant Pathol..

[4] Espada M., Silva A.C., Eves-van den Akker S., Cock P.J.A., Mota M., Jones J.T. (2016). Identification and characterization of parasitism genes from the pinewood nematode *Burspahelenchus xylophilus* reveals a multilayered detoxification strategy. Mol. Plant Pathol..

